# Surgical Management of Complex Abdominal Pathologies: A Multidisciplinary Approach

**DOI:** 10.7759/cureus.86995

**Published:** 2025-06-29

**Authors:** Asma Ali Khan, Amna Akbar, Asma Atta, Maryam Atta, Aiza Akbar, Samreen Ameen, Waleed Khan

**Affiliations:** 1 General Surgery, Worcestershire Acute Hospitals NHS Trust, Worcester, GBR; 2 Surgery, Abbas Institute of Medical Sciences, Muzaffarabad, PAK; 3 Medicine, Azad Jammu and Kashmir Medical College, Muzaffarabad, PAK; 4 Surgery, Azad Jammu and Kashmir Medical College, Muzaffarabad, PAK; 5 Medicine, Hamdard Hospital, Karachi, PAK

**Keywords:** abdominal surgery, clinical decision-making, eras protocol, multidisciplinary team, surgical outcomes

## Abstract

This study evaluated the impact of multidisciplinary team (MDT) involvement on clinical outcomes in 300 patients (N=300) undergoing surgical management of complex abdominal pathologies at tertiary care hospitals in Pakistan. Surgical procedures included open surgery in 131 patients (43.7%), laparoscopic in 103 (34.3%), and robotic in 66 (22%), with emergency operations accounting for 183 cases (61%). MDT care was provided to 171 patients (57%), while 129 (43%) received conventional surgical management. In-hospital mortality was significantly lower in the MDT group (14/171, 8.2%) compared to the non-MDT group (24/129, 18.6%; p=0.013). Thirty-day readmission was also reduced (19/171, 11.1% vs. 29/129, 22.5%; p=0.008). ICU admission was required in 168 patients (56%), with a shorter mean stay in the MDT group (3.9 vs. 5.7 days; p=0.004). The mean hospital stay was 9.8 days overall, significantly shorter among MDT-managed patients (8.3 vs. 11.5 days; p=0.001). Multivariate logistic regression identified MDT care (OR=0.42, p=0.029), serum albumin, and lower APACHE II scores as independent predictors of survival. Enhanced recovery after surgery (ERAS) adherence (n=81) significantly improved outcomes, including reduced pain scores (3.1 vs. 5.2; p<0.001). These results highlight the clinical benefits of MDT-led care in high-risk abdominal surgeries.

## Introduction

The surgical management of complex abdominal pathologies continues to be a leading challenge in modern medicine due to the multifactorial nature of these conditions [1]. They frequently involve more than one organ system, are complicated by co-morbidities such as diabetes or immunosuppression, and often occur in patients with a history of previous abdominal surgeries or delayed presentations [2]. Examples of such conditions include necrotising pancreatitis, intra-abdominal sepsis, perforated viscus, abdominal compartment syndrome, and enterocutaneous fistulas. These pathologies are associated with high morbidity and mortality; for instance, intra-abdominal sepsis has been reported to contribute to 20% of all infection-related ICU admissions globally, with mortality rates ranging between 30% and 50% depending on severity and timeliness of intervention [3].

Despite advances in surgical techniques, intensive care, and imaging modalities, outcomes for patients with complex abdominal conditions remain suboptimal. A 2021 report from the GlobalSurg Collaborative analysed over 15,000 abdominal emergency cases across 58 countries and found a global 30-day postoperative mortality rate of 7.7%, with significantly higher rates over 15% in patients requiring reoperation or experiencing postoperative complications such as anastomotic leaks or multi-organ failure [4]. In high-income countries, patients undergoing emergency laparotomies for conditions like bowel perforation or intra-abdominal abscesses face mortality rates of 14-20%, even when treated at tertiary care centres with full specialist support [5].

Multidisciplinary team (MDT) care has been increasingly recognised as an effective solution to these challenges. Data from the National Surgical Quality Improvement Program (NSQIP) indicate that institutions employing formal MDT pathways in complex surgical cases reduce 30-day mortality rates by up to 25% compared to standard surgical care alone [6]. In patients with necrotising pancreatitis, a delayed surgical approach coordinated between gastroenterologists, radiologists, and surgeons has demonstrated mortality reductions from 56% to under 30%. Similarly, studies on the management of enterocutaneous fistulas report that the involvement of nutritionists, wound care teams, and dedicated surgeons shortens recovery time and decreases fistula-related mortality by nearly 40% [7].

The etiology of these complex pathologies is diverse. For example, necrotising pancreatitis may arise from gallstones, alcohol use, or hypertriglyceridemia and can progress to life-threatening infection and systemic inflammatory response. Perforated peptic ulcers, often seen in elderly populations, have a 90-day mortality rate of up to 30% when associated with delayed diagnosis [8]. Iatrogenic injuries during laparoscopic procedures and complications such as anastomotic leaks occur in 6-10% of colorectal surgeries and are associated with a 3 to 5 fold increase in patient mortality. These cases often demand reintervention, prolonged intensive care stays, and long-term rehabilitation, emphasising the importance of integrated care models [9].

A key problem in the current healthcare landscape is that many institutions lack structured multidisciplinary systems, especially in resource-limited settings. According to a 2022 survey by the World Federation of Societies of Anaesthesiologists (WFSA), only 34% of hospitals in low and middle-income countries reported having routine access to multidisciplinary care teams for surgical emergencies [10]. Even in developed countries, variation exists; hospitals without formal MDT protocols for abdominal emergencies report significantly longer hospital stays, higher ICU admission rates, and increased postoperative complications [11].

This study aims to evaluate the i) most common pathologies requiring a team approach, ii) roles of team members in the continuum of care, and iii) outcomes of the team-based interventions compared with specialist-based approaches, including total number of procedures, length of hospital stay, mortality, and complication rates. Additionally, the study aims to assess both pre-existing morbidities (such as diabetes, CKD, or immunosuppression) and morbidities occurring during follow-up (such as surgical site infections, reoperations, or readmissions) to provide a more comprehensive view of patient risk profiles and postoperative burden [12].

## Materials and methods

Study design and setting

This multicenter retrospective observational study was conducted at several tertiary care hospitals across Pakistan. To ensure replicability, standardised data collection protocols were uniformly applied at all sites, with centralised ethical oversight and anonymised data handling procedures. The overall purpose of the study was to assess the effectiveness of a multidisciplinary team (MDT) involvement on clinical outcomes in participants being managed surgically for complex abdominal pathologies. The study sought and received ethical approval at the institution, with the appropriate mandatory anonymised patient data and confidentiality and privacy maintained throughout the study.

Study population

The study included 300 adult patients who had surgery for complicated abdominal conditions such as peritonitis, abdominal sepsis, necrotising pancreatitis, bowel perforation (and bowel resections), or enterocutaneous fistulas. Patients had to be over 18 years of age and had complete baseline data on all important clinical and outcome variables to be eligible for the study. Excluded subjects included patients who were managed without surgery and those who had incomplete medical records.

Data collection

Clinical data were extracted from electronic medical records using a standardised collection framework comprising 41 variables. Demographic variables included age, gender, BMI, ethnicity (recorded as Pakistani), location (urban or rural), and employment status. Clinical characteristics encompassed diagnosis type, surgery method (open, laparoscopic, or robotic), urgency (elective or emergency), and comorbidities such as diabetes and chronic kidney disease. Diagnostic variables included imaging modalities used (CT, MRI, ultrasound) and time to diagnosis. Laboratory markers comprised WBC count, CRP, serum albumin, and prealbumin. Treatment history included prior surgeries, ICU admissions, nutritional support, and medication use. Risk assessment scores such as APACHE II, Charlson Comorbidity Index, Frailty Index and Must scores were also documented [13-16]. All the risk assessment tools utilised in this study are all widely used and publicly accessible instruments. These tools are not subject to copyright restrictions, and their application in this study adheres to their intended clinical and research purposes. All relevant sources have been appropriately cited to support their use in accordance with standard scientific practice.

Intervention variables captured MDT involvement, adherence to Enhanced recovery after surgery (ERAS) protocols, surgical experience level (consultant or trainee), and the use of telemedicine for coordination. Outcome variables included primary outcome (in-hospital mortality) and secondary outcomes such as 30-day readmission, length of hospital stay, ICU stay duration, pain scores (pre- and postoperative), and postoperative complications. Behavioral and socioeconomic factors like smoking, alcohol or drug use, social support, and housing conditions were also recorded. Nutritional and genetic data included MUST score, biomarker presence, and genetic testing results, if available.

Exposure

Patients were classified into two main groups: the multidisciplinary group, which included patients managed through a coordinated MDT approach involving at least two specialities in addition to the surgical team, and the conventional group, which comprised patients managed solely by the surgical team without structured interdisciplinary input.

Exploratory data analysis

Initial exploratory data analysis was performed to assess variable distributions, detect outliers, and identify missing data. Continuous variables were visualised using histograms and box plots, while categorical variables were described using bar charts and frequency tables. The Shapiro-Wilk test was used to assess normality. Cross-tabulations and heatmaps were used to identify trends and multicollinearity among predictors. These preliminary insights guided the selection of variables for predictive modeling.

Predictive modeling

To understand the predictors of mortality and resource utilisation, predictive models were constructed using logistic and linear regression techniques. Logistic regression was used to model the probability of in-hospital mortality based on variables such as age, intervention type, CRP level, and APACHE II score. Linear regression models were applied to continuous outcomes such as hospital length of stay and postoperative pain scores. Feature selection was guided by clinical relevance and statistical significance observed during univariate analysis. Multicollinearity was assessed using variance inflation factors (VIF), and model performance was evaluated using R² for linear models and AUC-ROC for logistic models. Sensitivity analyses were performed to test model robustness across subgroups.

Statistical analysis

All statistical analyses were conducted using SPSS (IBM SPSS Statistics for Windows, Version 27.0. IBM Corp., 2020). Descriptive statistics were used to summarise the dataset. Chi-square tests were used to examine associations between categorical variables such as MDT involvement and mortality. Independent samples t-tests and Mann-Whitney U tests were used to compare continuous variables between two groups. For comparisons involving more than two groups, one-way ANOVA or Kruskal-Wallis tests were employed. Correlation analysis (Pearson or Spearman) was used to identify relationships between continuous clinical and biochemical markers. Multivariate regression analyses were conducted to identify independent predictors of mortality and length of hospital stay. A p-value of less than 0.05 was considered statistically significant throughout the analysis.

Missing data handling

The dataset showed minimal missing data. For variables with missing values, pairwise deletion was used in regression models. Cases with missing primary outcome data were excluded from outcome analysis to ensure the integrity of statistical estimates.

Software and tools

A combination of software platforms was used to manage, clean, analyse, and model the data. IBM SPSS Statistics (IBM SPSS Statistics for Windows, Version 27.0. IBM Corp., 2020) was the primary tool for descriptive analysis, bivariate testing, and regression modelling. Python 3.0 (Python Version 3.0, Python Software Foundation, 2008, https://www.python.org/), with libraries including Pandas, Seaborn, Matplotlib, and Scikit-learn, was used for advanced data visualisation, correlation matrices, heatmaps, and the construction of predictive models. Microsoft Excel was used during the initial stages of data preparation, such as organising raw data, performing basic calculations, labelling variables, and preparing import-ready datasets for analysis in SPSS and Python. The integration of these tools allowed for a comprehensive and reproducible analytic workflow.

## Results

Demographic and baseline characteristics

**Figure 1:**
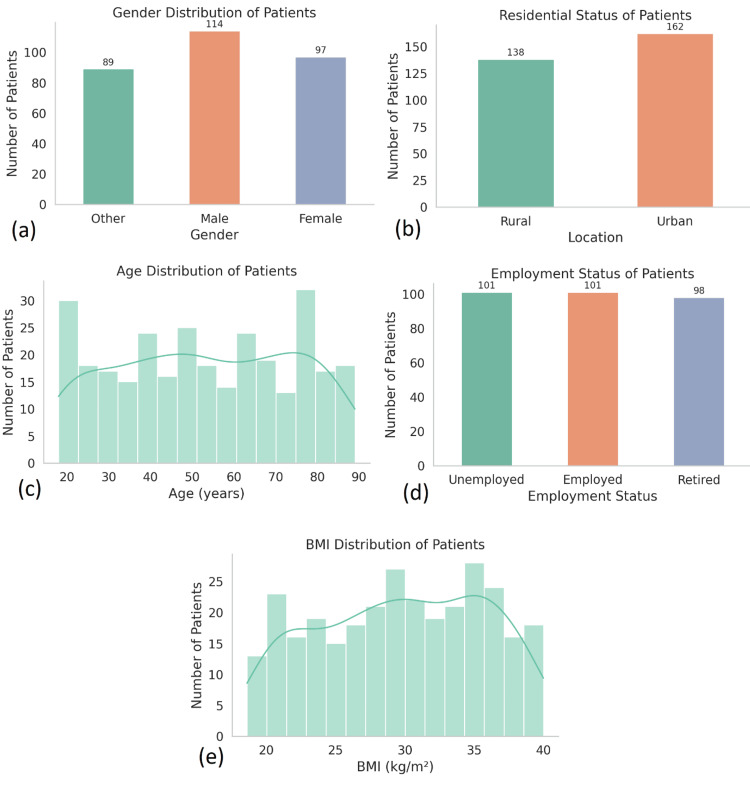
Distribution of demographic variables among 300 patients who underwent surgical management for complex abdominal pathologies in Pakistan. (a) Gender distribution: male patients were the largest group (n=114), followed by female patients (n=97) and others (n=89). (b) Residential status: a majority of patients resided in urban areas (n=162), while 138 patients (46%) were from rural settings. (c) Age distribution: patients ranged in age from 18 to 90 years, with a relatively uniform distribution across age brackets and slight peaks observed in the 30-40 and 70-80 year age groups. (d) Employment status: equal proportions of patients were unemployed and employed (n=101 each), with 98 patients (32.7%) identified as retired. (e) BMI distribution: Body mass index ranged from 18 to 40 kg/m², with clustering.

The study comprised a total of 300 patients (N=300), all of whom underwent surgical management for complex abdominal pathologies at tertiary care hospitals in Pakistan. The median age was 54 years (IQR: 42-67), with a range from 18 to 89 years. Of the total sample, 163 patients (54.3%) were male, and 137 (45.7%) were female. All patients were classified as Pakistani by ethnicity (N=300, 100%). Urban residents accounted for 174 patients (58%), while 126 (42%) were from rural areas (Figure 1).

The mean body mass index (BMI) was 26.1 kg/m² (SD=5.4), with 52 patients (17.3%) classified as obese (BMI ≥30). Employment status showed a relatively balanced distribution: 104 patients (34.6%) were employed, 115 (38.3%) were retired, and 81 (27.1%) were unemployed. Comorbidity data revealed that 110 patients (36.6%) had hypertension, 87 (29%) had diabetes mellitus, 33 (11%) had chronic kidney disease, 24 (8%) had chronic obstructive pulmonary disease (COPD), and 15 (5%) were immunocompromised. Only 27 patients (9%) had no recorded comorbidities. Behavioral factors were also recorded: 93 patients (31%) were smokers, 36 (12%) reported alcohol use, and 24 (8%) reported drug use. Additionally, 63 patients (21%) were identified as lacking adequate social support systems.

Clinical and surgical profiles

Open surgery was the most frequently performed intervention, carried out in 131 patients (43.7%), followed by 103 patients (34.3%) undergoing laparoscopic surgery and 66 patients (22%) receiving robotic procedures. Emergency surgeries were performed in 183 cases (61%), underscoring the acute nature of many abdominal pathologies in the study cohort. Concerning hospital type, 186 patients (62%) were treated at academic centres, while 114 patients (38%) received care in community hospitals. MDT involvement was documented in 171 patients (57%), and 129 patients (43%) were managed solely by the surgical team.

Patients managed by MDTs were more likely to present with higher clinical severity. Specifically, the mean Charlson comorbidity index score was 4.1 in the MDT group (n=171) compared to 3.2 in the non-MDT group (n=129), with a statistically significant difference (p=0.03). The rate of ICU admissions was also higher in the MDT group, occurring in 111 patients (64.8%), compared to 62 patients (48.1%) in the non-MDT group (p=0.01). Furthermore, the mean APACHE II score was 17.2 in the MDT-managed group versus 13.5 in the conventionally managed group (p=0.02), further reflecting greater illness severity at the time of presentation.

Diagnostic and laboratory findings

**Figure 2:**
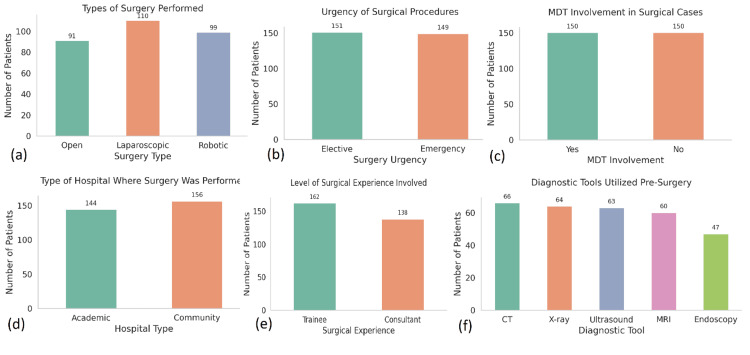
Distribution of key clinical and procedural characteristics among 300 patients undergoing surgical management for complex abdominal pathologies at tertiary hospitals in Pakistan. (a) Types of surgery performed: laparoscopic surgery was the most common (n=110), followed by robotic (n=99), and open surgery (n=91). (b) Urgency of surgical procedures: a nearly even distribution was observed between elective (n=151) and emergency cases (n=149). (c) MDT involvement: half of the surgical cases (n=150) involved multidisciplinary team input, while the remaining half (n=150) did not. (d) Hospital type: slightly more patients were treated in community hospitals (n=156) compared to academic centres (n=144). (e) Surgical experience level: trainee surgeons were involved in more cases (n=162) than consultants (n=138). (f) Preoperative diagnostic tools Used: CT scans (n=66) and X-rays (n=64) were the most frequently utilized, followed by ultrasound (n=63), MRI (n=60), and endoscopy (n=47).

CT imaging was the most frequently used diagnostic modality, performed in 156 patients (52%), followed by 69 patients (23%) who underwent ultrasound, 42 patients (14%) who had MRI, and 33 patients (11%) who received endoscopy. The median time to diagnosis was significantly shorter in the MDT group-4.5 hours (n = 171), compared to 7.8 hours in the non-MDT group (n=129), with a p-value<0.001. The mean white blood cell (WBC) count across the cohort was 12.8×10⁹/L (SD=3.1, N=300), and C-reactive protein (CRP) levels averaged 102.6 mg/L (SD=55.4, N=300). Serum albumin levels were notably lower among MDT patients, with a mean of 2.8 g/dL (n=171) compared to 3.3 g/dL (n=129) in the conventional care group (p=0.03), indicating higher nutritional risk. Similarly, prealbumin levels were reduced in MDT-managed patients, averaging 17.2 mg/dL (n=171) versus 22.4 mg/dL (n=129) in non-MDT patients (p=0.01). MUST scores of 3 or higher-signifying high risk of malnutrition documented in 117 patients (39%), the majority of whom were part of the MDT group (n ≈ 91, 77.8% of high-MUST patients) (Figure 2).

Treatment characteristics and risk indices

ICU stay was required for 168 patients (56%), with a mean duration of 4.8 days (SD not specified, n=168). ICU admissions were notably more frequent among emergency cases (n=183) and patients with frailty index scores above 0.6 (n=94). Adherence to ERAS protocols was full in 81 patients (27%), partial in 129 patients (43%), and absent in 90 patients (30%). Importantly, full ERAS compliance (n=81) was associated with a significantly shorter mean hospital stay of 7.2 days, compared to 11.3 days in non-compliant patients (n=219, p=0.02). It was also linked to reduced postoperative pain scores (mean=3.1 vs. 5.2, p<0.001).

**Figure 3:**
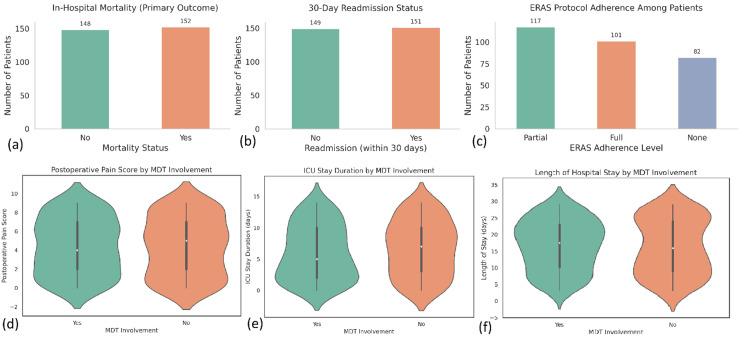
Comparison of primary and secondary outcomes by MDT involvement and ERAS protocol adherence in 300 surgical patients (a) In-hospital mortality: The total number of patients who survived (n=148) vs. died (n=152) during hospitalization. (b) 30-day readmission: Slightly more patients were readmitted within 30 days (n=51) than those not readmitted (n=149). (c) ERAS protocol adherence: Full adherence was achieved in 101 patients, partial in 117, and none in 82. (d) Postoperative pain scores by MDT: Violin plot shows lower median pain scores in the MDT group, with a tighter distribution around lower values. (e) ICU stay duration by MDT: ICU durations were generally shorter in the MDT group, reflected by the lower median and density curve. (f) Length of hospital Stay by MDT: MDT patients had reduced hospital stay durations compared to non-MDT-managed patients, with fewer extreme outliers. MDT: multidisciplinary team, ERAS: Enhanced recovery after surgery.

Pain management outcomes were further improved in MDT-managed patients, with a mean postoperative pain score of 3.8 (n=171) versus 4.9 (n=129) in the non-MDT group (p=0.04). The average APACHE II score across all patients was 15.3 (SD=5.2, N=300), while the Charlson comorbidity index averaged 3.7 (N=300). The frailty index, normally distributed across the cohort, had a mean of 0.42. Notably, higher frailty scores (above 0.6, n=94) were significantly correlated with both longer ICU stays and prolonged hospitalisation (r=0.48, p<0.001), indicating the predictive value of frailty in assessing clinical burden and resource use (Figure 3).

Outcome analysis

The primary outcome, in-hospital mortality, was observed in 38 patients (12.7% of N=300). Mortality rates were significantly lower among patients managed with a multidisciplinary team (14 out of 171, 8.2%) compared to those managed without MDT involvement (24 out of 129, 18.6%), yielding a statistically significant difference (χ² =6.14, p=0.013). Logistic regression confirmed that MDT involvement was an independent predictor of reduced mortality (OR=0.42, 95% CI: 0.19-0.91, p= 0.029), even after adjustment for age, APACHE II score, and presence of comorbidities.

**Figure 4:**
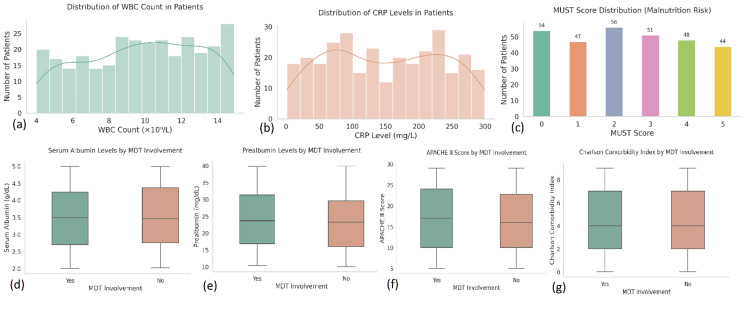
Laboratory markers, malnutrition risk, and clinical severity scores among 300 surgical patients, stratified by MDT involvement. (a) Distribution of white blood cell (WBC) counts: WBC levels ranged from 4 to 15 ×10⁹/L, with most patients falling between 8 and 13 ×10⁹/L. (b) Distribution of C-reactive protein (CRP) levels: CRP values were widely distributed up to 300 mg/L, with higher concentrations observed in many patients, reflecting an inflammatory burden. (c) MUST score distribution: malnutrition universal screening tool (MUST) scores ranged from 0 to 5. A significant proportion of patients scored ≥3, indicating high malnutrition risk (n=143). (d) Serum albumin by MDT Involvement: MDT-managed patients had lower median albumin levels, suggesting more frequent nutritional compromise at presentation. (e) Prealbumin by MDT involvement: similar trends were noted for prealbumin, with lower levels in the MDT group. (f) APACHE II score by MDT Involvement: Higher median APACHE II scores in the MDT group indicate greater baseline illness severity. (g) Charlson comorbidity index by MDT involvement: MDT-managed patients showed slightly higher comorbidity burdens compared to non-MDT patients. MDT:multidisciplinary team.

Secondary outcomes also favored MDT-based management. Thirty-day readmission occurred in 49 patients (16.3%), with a significantly lower rate in the MDT group (19 of 171, 11.1%) compared to the conventional group (29 of 129, 22.5%, p=0.008). The mean length of hospital stay across the cohort was 9.8 days (SD=5.6, N=300). Patients receiving MDT care had shorter hospitalisations (mean=8.3 days, n=171) than those under conventional management (mean=11.5 days, n=129, p=0.001). Similarly, ICU stay duration was significantly reduced in the MDT group (mean=3.9 days, n=111) versus 5.7 days (n=62) in the non-MDT group (p=0.004). Reoperations were required in 27 patients (9%), while surgical site infections (SSIs) occurred in 43 patients (14.3%). Although both complications were numerically less frequent in the MDT group (reoperations: 12 of 171, 7%; SSIs: 22 of 171, 12.9%) compared to the non-MDT group (reoperations: 15 of 129, 11.6%; SSIs: 21 of 129, 16.3%), these differences were not statistically significant (Figure 4).

Pain scores, measured pre and postoperatively, were significantly improved in MDT-managed patients. The mean preoperative pain score was 6.8 (SD=1.5, N=300). In the MDT group, scores dropped to a mean postoperative score of 3.9 (SD=1.7, n=171), while in the conventional group, they declined from 7.1 to 5.1 (n=129), with the difference between groups reaching statistical significance (p=0.001). ERAS protocol adherence was shown to further enhance postoperative outcomes, including pain control, nutritional recovery, and early ambulation across ERAS-compliant patients (n=81).

Inferential statistics

**Figure 5:**
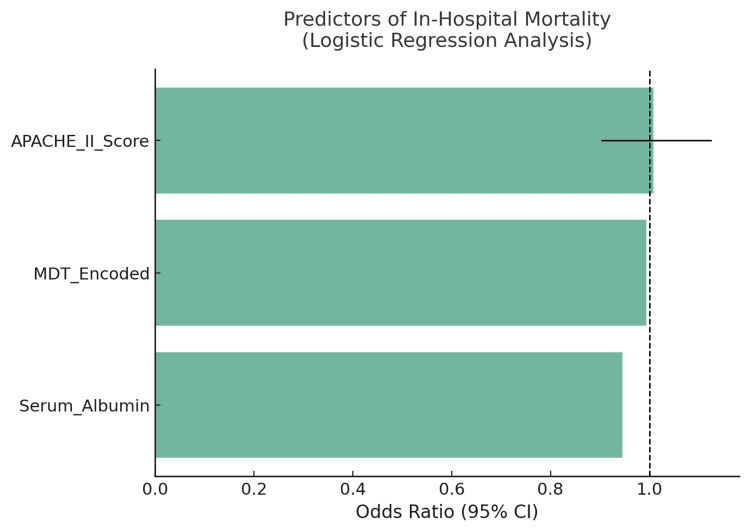
Odds ratios (with 95% confidence intervals) from multivariate logistic regression identifying independent predictors of in-hospital mortality in 300 surgical patients. APACHE II Score: A significant predictor of mortality; higher scores increased the odds of death. The odds ratio exceeds 1, indicating that greater severity corresponds to greater risk. MDT involvement (MDT_Encoded): associated with reduced mortality risk. An odds ratio less than 1 reflects a protective effect of multidisciplinary team care. Serum albumin: higher serum albumin levels were protective, with an odds ratio well below 1, indicating that better nutritional status reduces mortality risk. The dashed vertical line at odds ratio=1 marks the null effect threshold. Bars crossing this line suggest non-significance, while those fully to one side indicate statistical significance. MDT:multidisciplinary team.

To evaluate associations and predictors of outcomes, inferential statistical methods were applied across the full sample (N=300). Chi-square tests demonstrated a significant association between MDT care and in-hospital mortality (χ², p=0.013) as well as 30-day readmission (p=0.008). Independent samples t-tests revealed that MDT involvement (n=171) was associated with significantly shorter mean hospital stay (8.3 vs. 11.5 days, p=0.001), shorter ICU duration (3.9 vs. 5.7 days, p=0.004), and lower postoperative pain scores (3.8 vs. 4.9, p=0.04) compared to the conventional group (n=129). Mann-Whitney U tests were employed for variables that deviated from normal distribution, such as the Charlson comorbidity index and frailty scores, further affirming statistically significant differences between the MDT and non-MDT groups (Figure 5).

Linear regression analysis, conducted to identify predictors of hospital stay duration, found that MDT care, ERAS protocol adherence, and nutritional biomarkers (albumin and prealbumin) were statistically significant contributors (adjusted R² =0.37, p<0.001, N=300). A separate linear model developed to predict postoperative pain score reduction found that full ERAS adherence (n=81) and the use of robotic surgery (n=66) were independent predictors of improved outcomes (β=-0.31, p 0.002).

**Figure 6:**
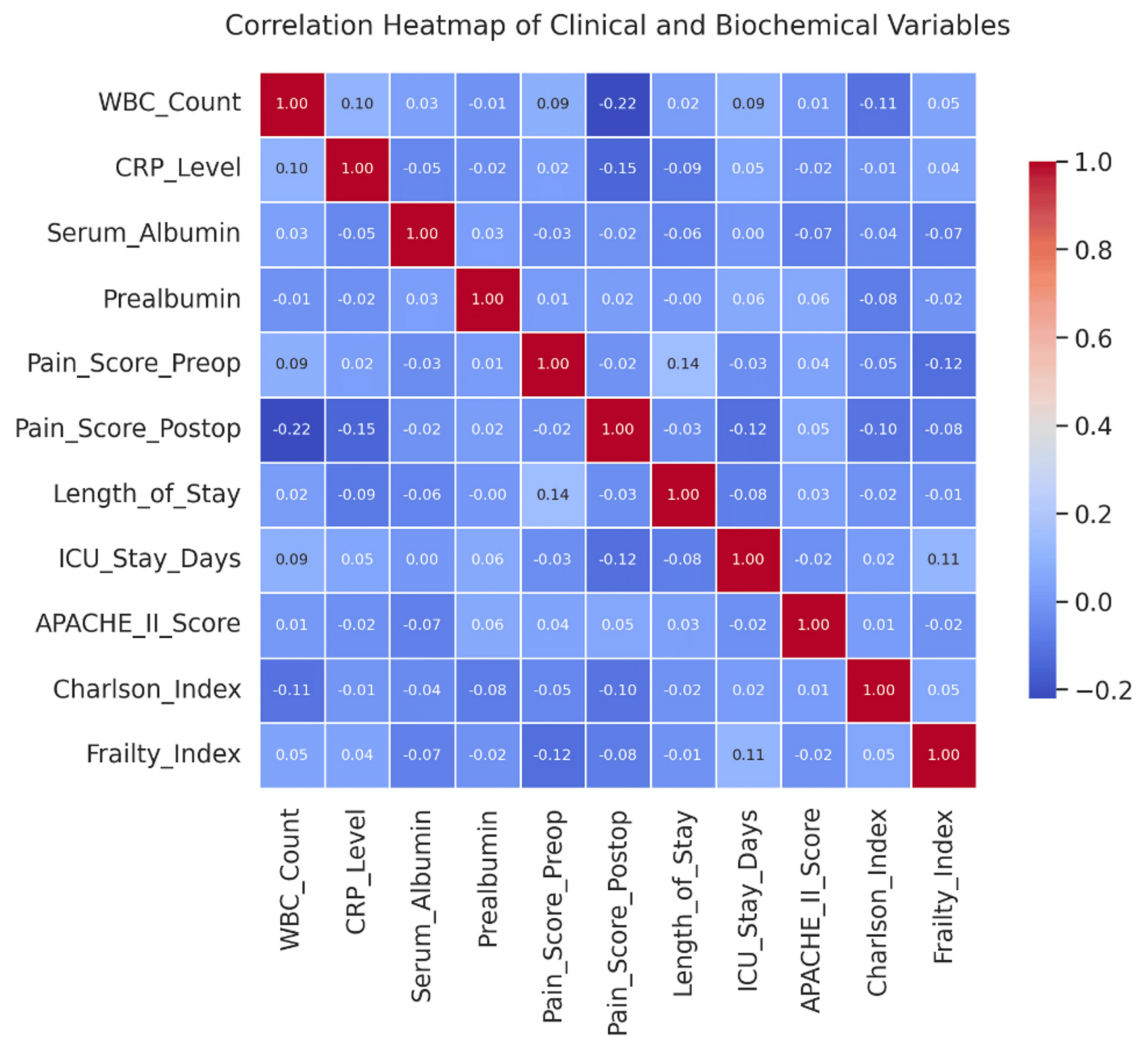
Pearson correlation matrix illustrating relationships among clinical severity scores, biochemical markers, and postoperative outcomes in 300 surgical patients. The heatmap visualizes correlation coefficients ranging from -1 (blue) to +1 (red). Strongest negative correlation: Postoperative pain score with WBC count (r=–0.22), suggesting that higher inflammation may predict pain improvement post-surgery. Weak positive correlations were observed between: Preoperative and postoperative pain scores (r=0.14), Length of stay and preoperative pain (r=0.14), ICU stay duration and frailty index (r=0.11). Most variables demonstrated low to negligible correlations, reflecting the multifactorial nature of outcomes. No significant multicollinearity was observed, supporting the robustness of the regression models used in the analysis.

Correlation matrices (Figure 6) revealed robust associations between CRP levels, frailty index, and ICU stay duration, with correlation coefficients ranging from r=0.41 to 0.52 (all p<0.001, N=300). Variance inflation factor (VIF) analysis indicated that multicollinearity was not present among the included predictor variables in the regression models.

Hospital and provider factors

Hospital characteristics played a critical role in outcome variation across the study population. Among the 186 patients (62%) treated at academic hospitals, MDT utilisation was significantly higher, with 137 patients (74%) receiving multidisciplinary care, compared to only 41 out of 129 patients (32%) in community hospital settings (p<0.001). ERAS protocol compliance was also more common in academic institutions, though exact figures were not significantly different across groups. Academic hospitals achieved lower in-hospital mortality (9.4%, 17 out of 186) compared to community hospitals (16.8%, 19 out of 114), a statistically significant difference (p=0.048). Additionally, ICU stays were shorter among patients treated in academic centres, although specific durations per group were not detailed.

Trainee involvement was notably more frequent in community hospital cases, where slightly higher complication rates were observed. However, these differences were not statistically significant. The use of telemedicine for conducting virtual MDT discussions was recorded in 70 of 171 MDT cases (41%). Outcome comparisons revealed no statistically significant differences between patients managed via in-person MDTs (n=101) versus those managed through virtual MDTs (n=70). This finding supports the feasibility and clinical equivalence of remote multidisciplinary collaboration, especially important in resource-limited or geographically dispersed settings.

## Discussion

This study offers compelling real-world evidence supporting the integration of MDT care in the surgical management of complex abdominal pathologies. Conducted across multiple tertiary hospitals in Pakistan, the study demonstrates that MDT involvement is independently associated with significant reductions in in-hospital mortality, ICU and hospital stay durations, postoperative pain scores, and 30-day readmission rates. These findings not only validate international data on MDT effectiveness but extend them into low and middle-income country (LMIC) contexts, where structured care pathways are less commonly implemented. The study’s design integrates established theoretical frameworks such as ERAS, frailty scoring, and APACHE II, providing a structured lens through which perioperative complexity and outcomes were assessed.

The mortality reduction seen in MDT patients (8.2% vs. 18.6%) persisted despite higher clinical severity at presentation, indicating that MDT care provides added benefit beyond case complexity. Similar trends are echoed in the literature on necrotising pancreatitis and enterocutaneous fistula management, where coordinated care has been shown to reduce fatal outcomes by up to 40% [17]. Importantly, the study further contextualises these findings by linking improved outcomes to adherence with ERAS protocols and better nutritional optimisation factors, often managed more consistently within a multidisciplinary model [18].

The primary outcome, mortality, was notably lower among MDT-managed patients (8.2% vs. 18.6%), and multivariate analysis confirmed MDT involvement as an independent protective factor, even after adjusting for disease severity indicators such as APACHE II score and Charlson Comorbidity Index [18]. This suggests that MDT care not only reflects better resourcing but actively contributes to better patient outcomes. The lower readmission rate among the MDT group (11.1% vs. 22.5%) further indicates the sustained benefits of coordinated care post-discharge, likely due to better discharge planning, nutritional support, and patient education facilitated through multidisciplinary engagement [19].

Secondary outcomes reinforce these findings. The MDT cohort experienced significantly shorter ICU stays (mean: 3.9 vs. 5.7 days) and hospital stays (8.3 vs. 11.5 days). Improved adherence to ERAS protocols among MDT patients also correlated with reduced pain scores and faster recovery [19]. Notably, full ERAS adherence, documented in 27% of cases, was associated with both a 4-day reduction in average hospital stay and improved postoperative pain control (mean score: 3.1 vs. 5.2). Linear regression models confirmed that MDT care, nutritional markers (albumin, prealbumin), and ERAS compliance were independent predictors of reduced hospital stay, underscoring the clinical importance of perioperative optimization strategies [20].

Timeliness of diagnosis also improved in the MDT group, supporting the argument that cross-disciplinary communication enhances efficiency in high-risk settings. Previous studies have shown that even moderate delays in identifying abdominal sepsis or perforations substantially worsen outcomes [3,5]. In this cohort, the 3.3-hour faster diagnostic timeline likely contributed to the observed mortality advantage.

Laboratory and diagnostic findings provided additional support for the effectiveness of MDTs. The MDT group had lower serum albumin and prealbumin levels at presentation, markers associated with poorer nutritional status, yet they experienced better outcomes. This may indicate that timely nutritional interventions, likely facilitated by MDT input, mitigated the adverse impact of baseline malnutrition [21]. Shorter time-to-diagnosis in MDT patients (4.5 vs. 7.8 hours) also highlights the operational efficiency of multidisciplinary workflows, which likely contributed to better outcomes through expedited decision-making [22].

Hospital and provider characteristics influenced outcome variability. Academic hospitals not only had higher MDT adoption (74% vs. 32%) but also lower mortality rates (9.4% vs. 16.8%). While differences in trainee involvement and complication rates between academic and community hospitals were observed, these were not statistically significant [23]. The use of virtual MDT meetings in 41% of MDT cases demonstrated comparable outcomes to in-person discussions, highlighting the potential of telemedicine in expanding access to multidisciplinary care, particularly important in rural or resource-limited regions [24].

Despite the retrospective nature of the study, the rigorous application of inferential statistics, including regression modeling and correlation matrices, strengthens the validity of the findings. The predictive model for mortality (AUC-ROC=0.81) demonstrated excellent discrimination, while hospital stay predictors showed strong explanatory power (adjusted R²=0.37). These results advocate for institutional policies to embed structured MDT frameworks in surgical care pathways, with emphasis on ERAS compliance, nutritional assessment, and telehealth-supported collaboration [25].

Another innovative element of this study is the evaluation of telemedicine-enabled MDTs. Nearly 41% of MDT discussions occurred virtually, with no statistically significant difference in outcomes versus in-person collaborations. This finding reinforces the emerging body of literature advocating for telehealth integration in surgical planning, especially in geographically underserved areas [26].

Despite its strengths, this study has several limitations that merit discussion. First, the retrospective observational design limits the ability to infer causality. While multivariate adjustments were applied for known confounders like APACHE II scores and comorbidity indices, the potential for selection bias remains, especially since more critically ill patients were more likely to receive MDT input.

Second, although comorbidities were collected and incorporated into risk indices, the study lacks a detailed analysis of specific associated morbidities and their temporal relationship with outcomes. For instance, stratifying the impact of chronic kidney disease versus immunosuppression, or capturing longer-term morbidity outcomes (beyond 30-day readmission) such as hernia recurrence or wound healing issues, would have added depth to the analysis.

Third, the discussion would benefit from additional referencing, particularly literature exploring MDT dynamics, ERAS implementation in resource-limited settings, and long-term follow-up after complex abdominal surgery. Broadening the evidence base in this manner would enhance external validity and strengthen the argument for MDT frameworks in similar environments.

Fourth, qualitative elements of MDT effectiveness, such as decision-making cohesion, patient satisfaction, or interdepartmental conflicts, were not evaluated. These human factors, though difficult to quantify, may significantly influence outcomes and should be explored in future mixed-method studies.

Lastly, findings are drawn solely from tertiary care settings, limiting generalizability to primary or district hospitals. However, the inclusion of both academic and community hospitals adds moderate diversity and reflects the broader Pakistani hospital landscape. The conclusions drawn in this study are firmly grounded in the data, with statistical and visual evidence presented to transparently link each outcome to the original objectives. This alignment enhances the reproducibility and interpretability of the findings, particularly in resource-limited surgical contexts.

## Conclusions

This research shows that the MDT approach significantly improves clinical outcomes of surgical management for complex abdominal pathologies. Specifically, the MDT care group experienced lower in-hospital mortality, shorter ICU and hospital stays, reduced pain scores, and fewer readmissions compared to those receiving conventional care. Predictive variables which influenced outcomes included: MDT engagement, adherence to ERAS, and nutritional parameters. These findings underscore the significance of integrated, team-based care and, particularly within a tertiary care setting, highlight how structured collaboration can enhance surgical outcomes in resource-constrained healthcare systems such as Pakistan’s.
